# Journal of Cellular and Molecular Medicine: the Future

**DOI:** 10.1111/jcmm.13075

**Published:** 2017-01-25

**Authors:** Stefan N. Constantinescu

**Affiliations:** ^1^ BrusselsBelgium

The field of Cellular and Molecular Medicine strives to understand the molecular basis of cell physiology and pathology, as well as cellular responses to extracellular stimuli. It integrates molecular cell biology, basic biochemistry and genetics with virology, bacteriology, physiology/pathophysiology, pharmacology, clinical research and bioinformatics. Results from Cellular and Molecular Medicine, along with advances in Genomics and Proteomics, have enabled the development of Precision/Personalized Medicine.

A mechanistic understanding of cellular function and molecular interactions is also being used to monitor states of health (state of wellness) and disease, and major international effort has been invested in identifying pre‐disease states and in developing ways to prevent progression to overt disease. A continuum can be established in certain cases at the molecular and cellular level between physiology and pathology, with markers, thresholds and a new biology covering those states.

One word that captures the area of interest of Journal of Cellular and Molecular Medicine (JCMM) is ‘mechanism’. JCMM aims to publish original and exciting studies that provide a mechanistic understanding of physiological and pathological processes. The review process must analyse results from many experimental systems addressing a wide range of biological questions, and we aim to publish well‐executed, novel and original studies that influence progress in the field and have a long‐lasting impact.

Our review process relies on a group of distinguished Editorial Board Members and Associate Editors, each of which is an expert in their specific area. We have a large database of reviewers with solid credentials in their fields. We work hard to give each manuscript a fair chance, and we review each manuscript in detail in terms of the experimental work carried out, the reproducibility and interpretation of results and the significance of the work. We think individual observations can change the world, provided they are true and valid. We do not aim to rewrite every paper for the authors, nor do we plan their future experiments. We carefully review the merits of the work under analysis. The vast majority of our effort is focussed on primary research papers (especially original articles), but we have published, and will continue to publish, commissioned or spontaneously submitted reviews, provided they offer new information, novel perspectives and authors have previously contributed to the field.

Given the broad scope of the journal, and its impact among scientists and clinicians, a major priority is to strengthen the quality of the manuscripts. Like all journals, we need to protect our readers from plagiarism and from misleading papers. That is why we strive to ensure a very thorough and competent review process. We aim to be recognized as a journal that publishes seminal observations that are confirmed and used by others. In other words, we aim to be positively cited.

Fast publication is another priority, and we aim to make original observations available to the scientific community as fast as possible so they are of use to other groups in the field. That is why our team works hard to rapidly publish accepted manuscripts online (before they are grouped in formal monthly issues). We do not delay valid and positively reviewed manuscripts that report one novel finding by asking for more complete studies with extensive *in vivo* transgenic animal model validation. That is not to say that minimal or incomplete data are sufficient, reasonable validation is always required.

A journal that focuses on Cellular and Molecular Medicine cannot ignore major unresolved scientific challenges such as understanding the protein folding code, the differentiation code with respect to chromatin spatial structure and gene expression, the molecular basis of communication between stem and progenitor cells, and many others. Results and novel approaches towards tackling these major challenges are within the areas of interest of JCMM.

In the same vein, JCMM is interested in publishing studies investigating the mechanisms of action of therapeutics and the molecular and cellular effects of those therapeutics. Recent years have also seen major advances in protein therapeutics such as replacement cytokines and hormones, but also for example, humanized antibodies that are being used for the treatment of many diseases and other protein therapeutics, like single‐chain antibodies and diabodies. Developments in this area are as important as the fundamental questions, because novel observations using these agents can help our understanding of cellular functions and the manipulation of cellular functions for therapeutic purposes.

Recent years have led to the increased integration of the biomedical field with genomics and proteomics. The successful sequencing of the human genome and developments in bioinformatics allow rapid analysis of proteins and genes studied by mass spectrometry and next‐generation sequencing, respectively. We are in the age of mutations and disease where certain mutations explain the phenotype or disease progression. In general, the identification of mutations allowed a paradigm shift in diagnosis and prognosis in many pathological conditions. The unexpected finding that several disease‐associated (and causing) mutations can be found in healthy individuals with, for example, clonal haematopoiesis due to ageing, but without pathological features, opened up an entirely new area of investigation whereby mutations alone do not suffice to produce disease. We encourage submission of manuscripts from this research area, provided a mechanism is explored.

We target the journal at young and senior researchers in biomedical and clinical research and also aim to attract readers with a broad interest in scientific progress. That is why we will focus on making the manuscripts accessible to the wider scientific community and why we insist on authors obviously explaining their findings so that conceptual advances can be accessible to a broad readership.

In short, we will publish original mechanistic observations that are experimentally sound and have potential for a significant impact in medicine. This has been the cornerstone of the journal since its foundation by Professor Laurentiu M. Popescu, a scientist at the forefront of cellular physiology, cell biology and pharmacology, who guided the direction of this journal to publish original and potentially spectacular observations that could inspire others.

